# Morphology and Micromorphological Examination of Giant Subcutaneous Fibrosarcoma in Goats: Five Cases

**DOI:** 10.1155/crve/6704467

**Published:** 2026-06-08

**Authors:** Saroj Kumar Yadav, Sunil Yadav

**Affiliations:** ^1^ Arabian Oryx Sanctuary, Environment Authority of Oman, Nepal; ^2^ Raaz Veterinary Hospital and Research Centre, Janakpurdham, Nepal; ^3^ Department of Medicine and Surgery, Chattogram Veterinary and Animal Sciences University, Chattogram, Bangladesh, cvasu.ac.bd; ^4^ Department of Anatomy and Histology, Faculty of Veterinary Medicine, Gazipur Agricultural University, Gazipur, Bangladesh

**Keywords:** caprine, fibrosarcoma, histology, spindle cell, subcutaneous tumor

## Abstract

Veterinary literature states that subcutaneous fibrosarcoma may occasionally occur in the ear of a goat. Fibrosarcoma is characterized by spindle‐shaped fibroblast proliferation and collagen fiber synthesis. Fibrosarcoma should be considered when examining large, solid subcutaneous lumps in goat breeds, emphasizing the importance of gross and histological testing if there is limited facility availability in the lab for the correct diagnosis and therapy. The study started in January 2022 and will end in December 2024. During this period, only five goats were recorded, consisting of four from the Jamuna Pari breed and one from the Tota Puri breed. The study describes the five breeds and sexes of goats with left‐side ear giant tumors and the clinical, diagnostic, and pathological aspects of a large subcutaneous fibrosarcoma. All goats were subjected to physical exams and gross and histological tests for diagnosis. The animal had a huge, round, and elliptical mass in the subcutaneous tissue that was rapidly growing, firm, and nonencapsulated, with a varied pink–grey–yellow color upon dissection. Histological examination revealed densely packed spindle cells with interlacing bundles, cellular atypia, and mitotic activity consistent with fibrosarcoma. The infiltrative cancer precluded complete surgical resection. In this investigation, the ear tumor was removed completely. The present study suggests fibrosarcoma as a possible cause of enormous, solid tumors under the skin in several goat breeds, highlighting the necessity of histological evaluation for correct diagnosis and management. Early diagnosis and management increase the outcome and quality of life of animals during the entire surgical procedure.

## 1. Introduction

Malignant mesenchymal connective tissue tumors, known as fibrosarcomas, are characterized by the proliferation of immature fibroblasts, or indistinguishable anaplastic spindle cells, organized in an interlaced pattern. These tumors are commonly observed in various locations across different species, including both male and female animals and humans, but are exceedingly rare or giant in ruminants, particularly goats, due to their short lifespan and diverse utility [1, 2].

Giant subcutaneous fibrosarcoma in goats generally appears as a steadily growing lump that could be initially interpreted as a benign lesion [3]. They are usually seen in the skin, under the skin, or both. The appearance of these tumors varies based on the species, age, and underlying cause of the disease. Older cats and dogs [4] most commonly exhibit this type of tumor, although it can occur in any species.

The literature contains limited occurrences of the condition manifesting as a cutaneous plaque or nodule on the trunk, limbs, head, neck, vulva, or toe [2]. Although the exact cause of goat fibrosarcoma is unknown, other species have been linked to chronic inflammation, genetic abnormalities, exposure to toxins, and even prior injections or trauma [5]. Owing to their infrequency and vague clinical symptoms, histological analysis is used for diagnosis, and surgical excision is usually the course of treatment, but recurrence is a possibility [3].

The goat pinna is also extremely uncommon; only a few occurrences have been documented in one breed [1]. Large giant neoplasms include caprine cutaneous pinna fibrosarcoma. To the best of our knowledge, only a few single goats have been reported to have certain large‐sized cutaneous tumors. This research paper examines the physical attributes, weight, anatomical positioning, dimensions, and histological features of well‐differentiated fibrosarcoma in goats.

## 2. Material and Methods

### 2.1. Study Areas, Duration, and Animal Population

The study was carried out at SAQ Teaching Veterinary Hospital CVASU, from January 2022 to December 2024. The total number of goats was five, including four Jamuna Pari–bred and one Tota Puri–bred goat.

### 2.2. Surgical Preparation

**Figure 1:**
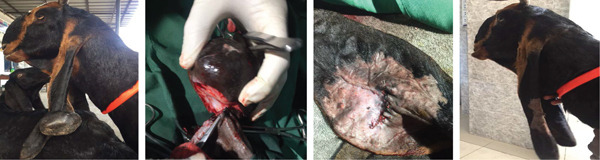
The surgical procedure for the correction of an ear tumor is sequential.

The standard surgical procedures were followed. After clipping and shaving, the surgical area was aseptically prepared with 10% povidone‐iodine and 70% alcohol. An intravenous sedative, diazepam (injection Easium, Opsonin Pharmaceuticals Ltd., BD), was administered to the goats at a dosage of 0.5 mg/kg. Additionally, ring block anesthesia was applied at the ear base using 2% lidocaine HCl at a dosage of 1 mL/cm^2^ (Injection Jasocaine, Jayson Pharmaceuticals Ltd., BD). The goats were positioned laterally for the procedure. The mass was completely excised from the proximal area of the pinna. Each vessel was individually ligated, and the incision was closed with horizontal mattress sutures using 1/0 nylon suture material, as shown in Figure 1.

### 2.3. Postoperative Care and Management

All goats received an intramuscular administration of a streptomycin and penicillin combination (Steptopen Vet, Reneta Laboratories Ltd., BD) at dosages of 10 mg/kg and 20000 IU/kg body weight, respectively, every 24 h until the fifth postoperative day. Meloxicam (M‐paint, ACI Laboratories Ltd., BD) was supplied subcutaneously at a dose of 0.5 mg/kg for 3 days, whereas pheniramine maleate (Hista‐Vet, ACI Pharmaceuticals Ltd., BD) was administered intramuscularly at a dosage of 1 mg/kg for five postoperative days [6]. The surgical skin sutures were removed on the seventh day postoperatively.

### 2.4. Gross Observation

Gross observation was done on all five goats based on physical appearance, shape, size, consistency, weight, side of location in ear, and color after dissection of tumors [3].

### 2.5. Histological Observation

In the subsequent step, the samples were promptly immersed in a 10% buffered formalin solution for histological preparation. The intact, complete tumors were excised and sectioned into small fragments. A gradient of alcohol concentrations was employed to dehydrate the tissue, which was subsequently purified using xylene and subsequently embedded in a paraffin block. The block was subsequently divided into segments with a thickness of 5 *μ*m. The slices were subsequently analyzed microscopically after being stained with hematoxylin and eosin, as per the methodology outlined in a recent research [1].

## 3. Results

### 3.1. Gross Examination

**Figure 2:**
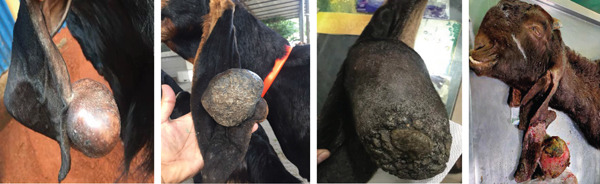
Images presenting the microscopic characteristics of tumors occurring in the left ear of various sexes and breeds of goats.

**Figure 3:**
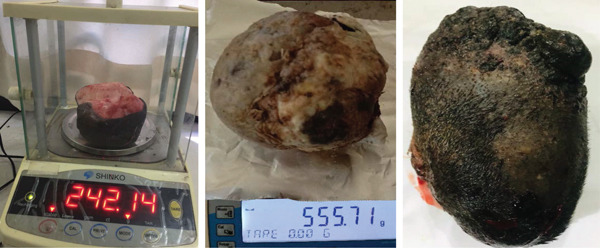
The gross observations of the tumor include round, rough, fibrous, and relatively nonuniform weight.

**Figure 4:**
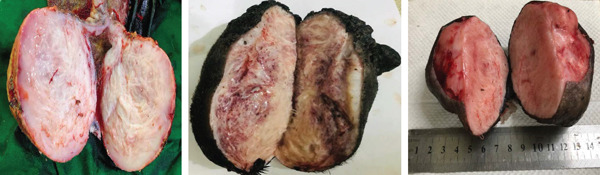
The tumor images exhibit a “pink–gray–yellow” coloration and “yellow–brown with abundant petechiae” and possess a “firm,” “smooth surface” in cross‐section.

The tip of the left ear tumor is the primary target region in all aged goats, and the Jamunapari female goat is more susceptible to tumors than the Tota Puri goat breed. The tumors’ mass, somewhat similar between the five tumors, which have a giant morphology, measures approximately 15 cm in length, 9 cm in diameter, and 4 cm in width. It showed up as a distinct, elongated, rough, spherical mass on the left pinna’s concave surface. Other than this lump, which was only partially covered by hairy skin, the animal displayed no clinical abnormalities (Figure 2). The mass did not feel uncomfortable to the touch, and there was no shaking of the head. Figure 3 shows their size and shape with somewhat similar weights. Upon cross‐sectioning, the surface color of the pale and light brown areas resembles a blood spot, exhibiting a half‐blackish hue with a cream color and a firm texture. Conversely, the opposite mass appears pale to pinkish with either a firm or rubbery nature, as illustrated in Figure 4. The mass exhibited clear boundaries that delineated it from the surrounding tissues without penetrating nearby structures. It was securely affixed to the inner surface of the pinna and exhibited a smooth texture. The neighboring tissues exhibited no indications of compression or ulceration. The bulk exhibited a markedly reduced number of hairs compared to the surrounding skin, which has a coarse texture. Table 1 reports information on the breeds, sexes, and tumor weights of the cases described here.

**Table 1:** Classification of goat tumors based on breed, age, sex, and weight.

Goats	Breed	Age (year)	Sex	Body weight (kg)	Located side tumors	Tumor type	Weight of tumors
1	Jamuna Pari	4	M (not castrated)	49	Left ear	Fibrosarcoma	242.14 g
2	Jamuna Pari	6	M (not castrated)	58	Left ear	Fibrosarcoma	321.30 g
3	Jamuna Pari	6	F	55	Left ear	Fibrosarcoma	555.71 g
4	Jamuna Pari	7	F	65	Left ear	Fibrosarcoma	1037.67 g
5	Tota Puri	8	F	45	Left ear	Fibrosarcoma	205.12 g

### 3.2. Histological Observation

**Figure 5:**
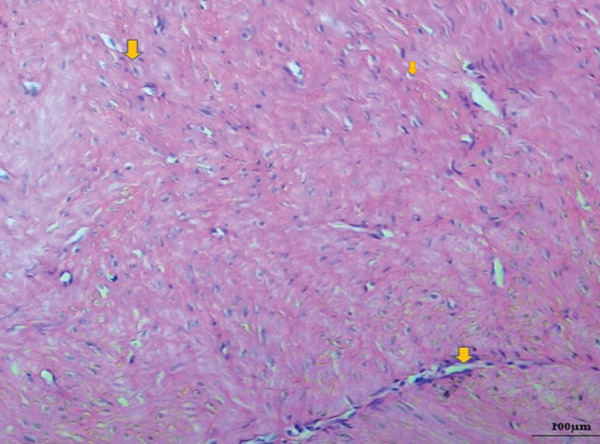
A representative slide from the examination of the tumors shows that spindle‐shaped tumor cells resembling fibroblasts are growing into the skin in a herringbone pattern at 100× magnification.

A cutaneous, expansive, focally infiltrative spindle cell tumor of intermediate cellular density was found by histopathologically analyzing the represented tissue samples at 100× (Figure 5). Most of the tumor cells were grouped in a herringbone pattern and looked like normal fibroblasts. The tumor cells occasionally had little pleomorphism, and the cytoplasm was scant. The nuclei showed slight differences in size and shape; they were mostly uniform, light in color, and stretched from oval to elongated with unclear nucleoli. Additionally, there were three mitoses observed in 10 randomly selected high‐power fields; however, multinucleated cells were absent. The tumor cells were identified within a limited quantity of collagenous matrix that exhibited positive staining with hematoxylin and eosin, and this stroma had a considerable number of blood vessels. Moderate, multifocal perivascular lymphocyte infiltration was also noted in the tumors. Furthermore, moderate edema and infiltration by neutrophilic granulocytes were observed in the superficial dermis. These results allow for the diagnosis of cutaneous low‐grade fibrosarcoma.

## 4. Discussion

Tumors of the subcutaneous tissue and skin are more prevalent in domestic animals [7]. The current study demonstrates that ear fibrosarcoma is exceptionally rare among different breeds and sexes in goats, as evidenced by [8]. The current study reveals that older female goats are more susceptible to tumors than males, a finding corroborated by [9]. The fact that males are routinely killed at an early age may be the reason for the increased frequency in females [10] observed that female domestic animals are more susceptible to cutaneous tumors than their male counterparts (56.5%). White‐haired goats are more likely to develop cutaneous tumors, according to [11]; however, current research indicates that black goats are more likely to develop tumors. Julia et al. [1] support the current study’s observation that the left ear is more prone to tumors. The current investigation found that fibrosarcomas continue to manifest as isolated masses, which is corroborated by [11]. While fine needle biopsies have been shown to yield less favorable results [12], the present investigation saw a similar outcome. The histopathological analysis of paraffin‐embedded, hematoxylin‐eosin‐stained specimens conducted in this investigation appears adequate for diagnosis by [9].

The present investigation established a final diagnosis of fibrosarcoma based on histological examination, corroborated by [13]. Based on the differentiating score, mitotic score, and tumors’ necrotic score, the tumors were classified as Grade 1 in accordance with the categorization method for cutaneous and subcutaneous soft tissue sarcoma in dogs [11]. This conclusion is due to the absence of a distinct grading system for tumors in ruminants by [1]. The size and location of the tumors dictate the symptoms of fibrosarcoma, which vary greatly. No clinical problems were observed aside from the mass due to the fibrosarcoma’s placement in the presented case.

Aggressive surgical excision is one therapy option for fibrosarcomas; nevertheless, euthanasia is often chosen for individuals with bone involvement when surgical intervention is impractical or unachievable. Despite reports of metastasis postsurgical intervention in cattle [14] and elevated recurrence rates in canines and felines [15], the reported goat showed no signs of metastases 10 months after surgery, nor any abnormal tumors in the ear, regional lymph nodes, or other body areas. The differential diagnosis should encompass other morphologically distinct soft tissue sarcomas, such as leiomyosarcoma, peripheral nerve sheath tumors, and perivascular wall tumors. It is possible to differentiate between these different kinds of soft tissue sarcoma using immunohistochemical characteristics. The present study has some limitations for immunochemistry due to the lack of available facilities in the laboratory, so better confirmation is needed, particularly through the use of advanced diagnostic techniques or collaboration with specialized laboratories that can provide the necessary resources.

## 5. Conclusions

In goats, subcutaneous fibrosarcoma is rare in Bangladesh and can affect any breed; however, it should be investigated when diagnosing fast‐developing, firm subcutaneous tumors, as histopathology is needed to confirm both clinical and gross findings.

## Funding

This study received SAQT Veterinary Hospital’s small fund for surgery support.

## Ethics Statement

For the objective of this research, goats were brought to the hospital for treatment purposes. Before the surgery, the SAQTV hospital authorities obtained the owner’s approval. In compliance with local laws and regulations, the research followed the protocols supplied by the ethics committee of Chattogram Veterinary and Animal Sciences University, Bangladesh, which oversaw the collection of animal samples and the execution of the studies.

## Conflicts of Interest

The authors declare no conflicts of interest.

## Data Availability

Data is available on request from the corresponding author.
